# Qualitative visual trichotomous assessment improves the value of fluorine-18 fluorodeoxyglucose positron emission tomography/computed tomography in predicting the prognosis of diffuse large B-cell lymphoma

**DOI:** 10.1186/s40880-015-0021-y

**Published:** 2015-06-10

**Authors:** Xu Zhang, Wei Fan, Ying-Ying Hu, Zhi-Ming Li, Zhong-Jun Xia, Xiao-Ping Lin, Ya-Rui Zhang, Pei-Yan Liang, Yuan-Hua Li

**Affiliations:** Sun Yat-sen University Cancer Center; State Key Laboratory of Oncology in South China, Collaborative Innovation Center for Cancer Medicine, Guangzhou, Guangdong 510060 P. R. China; Department of Nuclear Medicine, Sun Yat-sen University Cancer Center, Guangzhou, Guangdong 510060 P. R. China; Department of Hematological Oncology, Sun Yat-sen University Cancer Center, Guangzhou, Guangdong 510060 P. R. China; Department of Medical Oncology, Sun Yat-sen University Cancer Center, Guangzhou, Guangdong 510060 P. R. China

**Keywords:** Diffuse large B-cell lymphoma, Positron emission tomography/computed tomography (PET/CT), Prognosis, The qualitative visual trichotomous assessment (QVTA) criteria, The Deauville criteria

## Abstract

**Introduction:**

Fluorine-18 fluorodeoxyglucose (^18^ F-FDG) positron emission tomography/computed tomography (PET/CT) is a powerful tool for monitoring the response of diffuse large B-cell lymphoma (DLBCL) to therapy, but the criteria to interpret PET/CT results remain under debate. We investigated the value of post-treatment PET/CT in predicting the prognosis of DLBCL patients when interpreted according to qualitative visual trichotomous assessment (QVTA) criteria compared with the Deauville criteria.

**Methods:**

In this retrospective study, final PET/CT scans of DLBCL patients treated with rituximab-based regimens between October 2005 and November 2010 were interpreted using the Deauville and QVTA criteria. Survival curves were estimated using Kaplan-Meier analysis and compared using the log-rank test.

**Results:**

A total of 253 patients were enrolled. The interpretation according to the Deauville criteria revealed that 181 patients had negative PET/CT scan results and 72 had positive results. The 3 year overall survival (OS) rate was significantly higher in patients with negative scan results than in those with positive results (91.6 % vs. 57.5 %, *P* < 0.001). The 72 patients with positive scan results according to the Deauville criteria were divided into two groups by the interpretation according to the QVTA criteria: 29 had indeterminate results, and 43 had positive results. The 3 year OS rate was significantly higher in patients with indeterminate scan results than in those with positive results (91.2 % vs. 33.5 %, *P* < 0.001) but was similar between patients with negative and indeterminate scan results (91.6 % vs. 91.2 %, *P* = 0.921).

**Conclusions:**

Compared with the Deauville criteria, using the QVTA criteria for interpreting post-treatment PET/CT scans of DLBCL patients is likely to reduce the number of false positive results. The QVTA criteria are feasible for therapeutic outcome evaluation and can be used to guide risk-adapted therapy.

## Background

Diffuse large B-cell lymphoma (DLBCL) is the most common subtype of non-Hodgkin’s lymphoma, constituting 30 % to 40 % of all adult non-Hodgkin’s lymphoma cases in China. Despite attempts to increase the efficacy of conventional chemotherapy, treatment with rituximab, cyclophosphamide, doxorubicin, vincristine, and prednisone (R-CHOP) is not effective in approximately 40 % of DLBCL patients. The 5 year overall survival (OS) rates for patients with DLBCL vary from 45 % to 82 %, reflecting the heterogeneous nature of this disease [1, 2].

The response to treatment is an important outcome predictor for individual patients. Achieving complete remission after first-line chemotherapy is of paramount importance because it usually leads to longer survival, whereas an incomplete response is usually associated with a poorer outcome [3].

Fluorine-18 fluorodeoxyglucose (^18^ F-FDG) positron emission tomography/computed tomography (PET/CT) is a powerful tool both for primary staging and for monitoring the response to therapy in DLBCL and is used in the standardized response assessment criteria for lymphoma [4–6]. Qualitative visual dichotomous assessment is the most widely used method to assess the responses shown on PET/CT scans in the routine nuclear medicine setting and is the recommended method for post-treatment response evaluation in the revised criteria [5]. However, ^18^ F-FDG is not a highly specific marker; ^18^ F-FDG uptake after a few cycles of chemotherapy may stem from the presence of both persistent viable tumor and local inflammation [7], whereas a significant proportion of patients have prolonged survival in remission conditions despite positive PET/CT scans [8–10].

Here, we report the results of a retrospective study of patients newly diagnosed with DLBCL and treated with standard rituximab-based regimens, all of whom underwent ^18^ F-FDG PET/CT at the end of first-line chemotherapy. In an effort to standardize the interpretation of PET/CT scans, we evaluated the prognosis predictive value of final PET/CT (F-PET/CT) scans interpreted according to the qualitative visual trichotomous assessment (QVTA) criteria compared with the Deauville criteria.

## Methods

### Patient selection

This retrospective single-arm study enrolled adult patients with histologically proven, untreated DLBCL. The study was approved by the Sun Yat-sen University Cancer Center ethics committee. We retrieved and reviewed the clinical and follow-up records of patients who underwent F-PET/CT between 2005 and 2010. The data included age, sex, lactate dehydrogenase (LDH) level, international prognostic index (IPI) [11], extranodal involvement, bone marrow biopsy, full laboratory workup, echocardiography, the presence of relapse and death. Patients were staged according to the Ann Arbor staging system [7].

All patients were systematically followed every 3-6 months for the first 3 years after diagnosis and annually thereafter. At each follow-up time point, patients were either met at the clinic or contacted by phone to determine survival. Follow-up data were recorded at scheduled visits.

### ^18^ F-FDG PET/CT imaging

The patients were examined using a dedicated PET/CT system (Discovery ST-16, GE Health Care, Piscataway, NJ, USA). They were instructed to fast for 6 h and to abstain from caffeine and cigarettes for 24 h before the examination. ^18^ F-FDG (4.4-7.4 MBq/kg) was injected intravenously, and the patients were then requested to lie comfortably in a dark room for 60-90 min before the PET/CT scanning. The patients were scanned from the calves to the middle part of the femur while lying in a supine position. CT was performed before PET, and the resulting data were used to generate an attenuation correction map for PET. Two-dimensional PET images were reconstructed with a slice thickness of 3.25 mm using the ordered subset expectation maximization iterative image reconstruction method. PET, CT, and fused PET/CT images were generated for review on a Xeleris computer workstation.

### Visual analysis of PET/CT scans

Three nuclear medicine experts interpreted the PET/CT scans. Clinical information and baseline data were provided, but no follow-up information was given. Other causes of false positive scans were ruled out. Based on the reviewers’ experience, a more detailed set of instructions was established to resolve potential confounding variables. The panel agreed that at least two reviewers would assess the F-PET/CT scans of each patient.

All F-PET/CT images were analyzed using the Deauville 5-point scale: point 1, no residual uptake higher than the background level; point 2, residual uptake not higher than the mediastinum uptake level; point 3, residual uptake higher than the mediastinum uptake level but lower than the liver uptake level; point 4, residual uptake moderately increased compared with the liver uptake level; and point 5, residual uptake markedly increased compared with the uptake level of the liver or new sites of disease. The Deauville criteria were used for nodal and extranodal disease: scores of 1-3 were deemed negative; scores of 4 and 5 were deemed positive.

The QVTA criteria have the same negative criteria as the Deauville criteria, but different positive criteria. A score of 4 was defined as uptake higher than the liver uptake level and scattered and nonuniform ^18^ F-FDG distribution and was deemed indeterminate. A score of 5 was defined as uptake higher than the liver uptake level and centralized and uniform ^18^ F-FDG distribution or as new sites of disease and was deemed positive.

### Statistical analysis

Demographic and baseline disease characteristics were recorded, censoring the time of observation by November 2013. Because the objective of this analysis was to estimate the value of F-PET/CT in predicting the prognosis of DLBCL patients treated with standard rituximab-based regimens, the primary end point was OS. OS was defined as the time from the start of treatment to death of any cause or the last follow-up. The second end point was progression-free survival (PFS). PFS was defined as the time from the start of treatment to lymphoma progression, death of any cause, or the last follow-up. Estimates of survival were calculated according to the Kaplan-Meier method and compared with the log-rank test. A two-sided *P* value < 0.05 was considered significant. All statistical analyses were performed using SPSS version 16.0.

## Results

### Patient characteristics

The clinical characteristics of the 253 patients with histologically confirmed DLBCL are summarized in Table 1. The median age was 47 years (range, 18-79 years); 67 (26.5 %) patients were ≥ 60 years old. Sixty-five (25.7 %) patients had IPI scores indicating high-intermediate or high risk. Nearly half (49.8 %) of the patients had an Ann Arbor stage I or II tumor.

**Table 1:** Characteristics of the 253 patients with diffuse large B-cell lymphoma (DLBCL)

Variable	No. of patients [cases (%)]
Sex	
Men	137 (54.2)
Women	116 (45.8)
Ann Arbor stage	
I	48 (19.0)
II	78 (30.8)
III	58 (22.9)
IV	69 (27.3)
IPI score	
Low (0 or 1)	137 (54.1)
Low/intermediate (2)	51 (20.2)
High/intermediate (3)	44 (17.4)
High (4 or 5)	21(8.3)
Chemotherapy	
R-EPOCH	25 (9.9)
R-CHOP	228 (90.1)

*IPI*, international prognostic index; *R*, rituximab; *CHOP*, cyclophosphamide, doxorubicin, vincristine, prednisone; *EPOCH*, etoposide, prednisone, vincristine, cyclophosphamide, doxorubicin

### Treatment and outcome

Twenty-one (8.3 %) patients underwent surgery before chemotherapy. R-CHOP was the most commonly used primary chemotherapy regimen (90.1 %). Twenty-five (9.9 %) patients received rituximab, etoposide, prednisone, vincristine, cyclophosphamide, and doxorubicin (R-EPOCH) as the first-line chemotherapy regimen. The chemotherapy plan was changed for 9 (3.6 %) patients because of progression after 4 cycles of R-CHOP. Forty-five (17.8 %) patients received combined modality therapy with 4 cycles of R-CHOP or R-EPOCH followed by involved-field radiotherapy. All F-PET/CT scans were completed less than 3 months after the completion of primary treatment.

At a median follow-up of 47 months (range, 3-101 months), 54 (21.3 %) patients died of progressive disease (*n* = 41), infection (*n* = 5), heart failure (*n* = 2), renal failure (*n* = 1), lung cancer (*n* = 1), suicide (*n* = 1), or unknown causes (*n* = 3).

### PET/CT characteristics

**Fig. 1:**
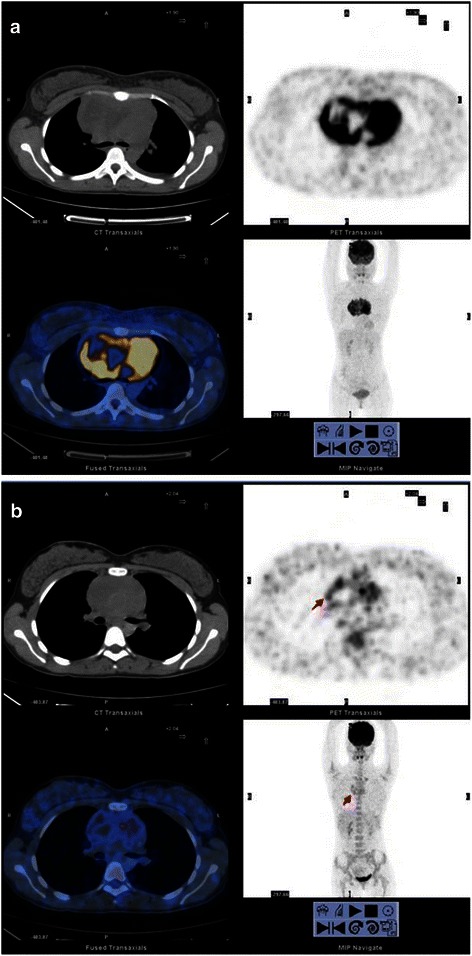
Fluorine-18 fluorodeoxyglucose (^18^ F-FDG) positron emission tomography/computed tomography (PET/CT) scans of diffuse large B-cell lymphoma (DLBCL) with a score of 4. (**a**), baseline PET/CT; (**b**), final PET/CT (F-PET/CT). A 26 year-old woman with bulky mediastinal uptake on baseline PET/CT, which was interpreted as indeterminate on F-PET/CT using the qualitative visual trichotomous assessment (QVTA) criteria; the residual foci (*red arrow*) were diagnosed and scored as 4. However, the Deauville criteria interpreted the scan as positive. The patient was alive and in complete remission 41 months after treatment initiation

**Fig. 2:**
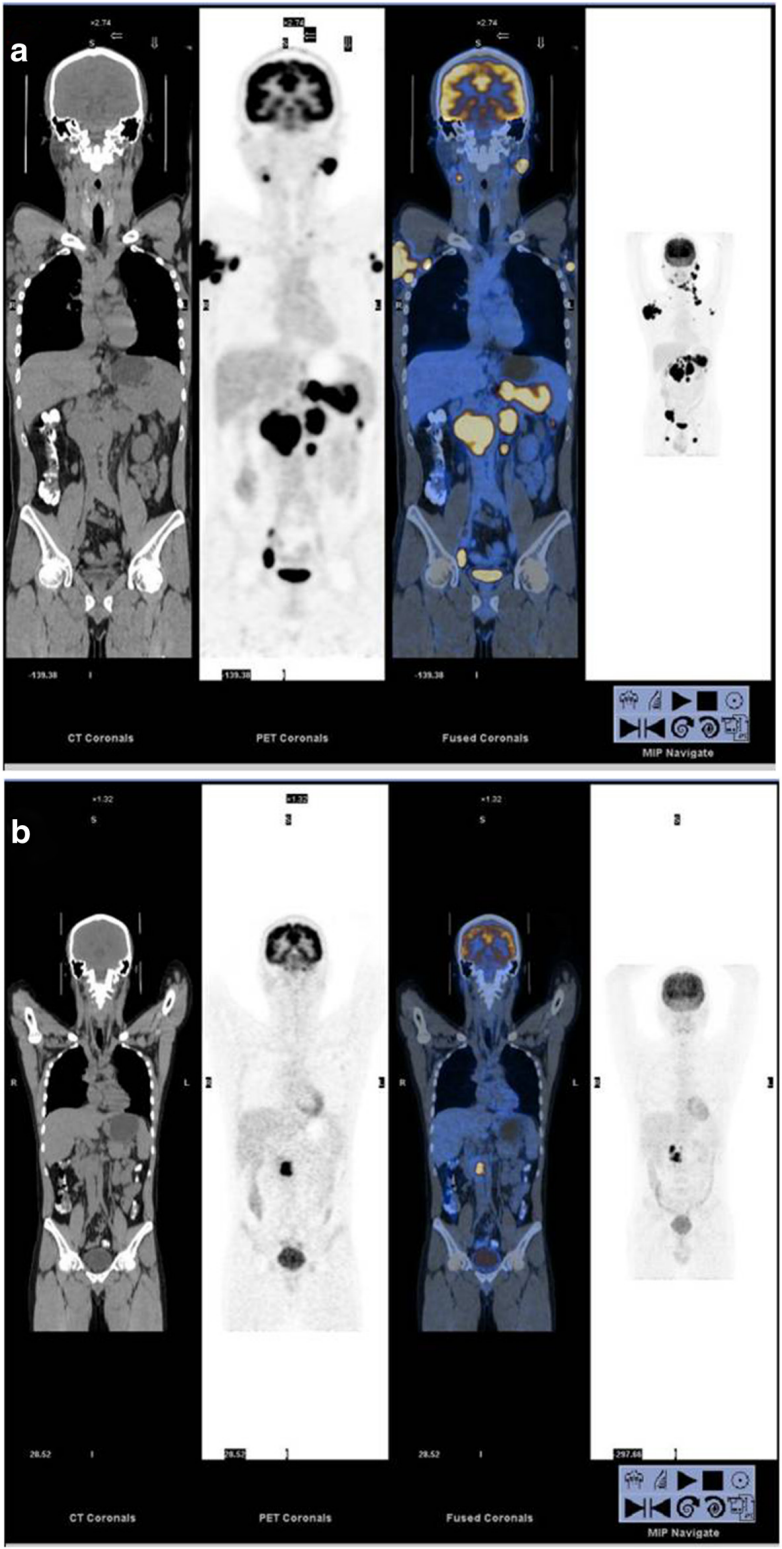
PET/CT scans of DLBCL with a score of 5 in a 45 year-old man. (**a**), baseline PET/CT; (**b**), F-PET/CT. Baseline PET/CT shows an Ann Arbor stage IV tumor, which was interpreted as positive on F-PET/CT using the QVTA criteria; the residual foci (*red arrow*) were diagnosed and scored as 5. This patient received involved-field radiotherapy after 6 cycles of chemotherapy using rituximab, cyclophosphamide, doxorubicin, vincristine, and prednisone (R-CHOP) regimen, but the disease progressed, and the patient died 31 months after treatment initiation

According to the Deauville criteria, 181 patients had negative F-PET/CT scan results and 72 had positive results. According to the QVTA criteria, the 72 positive patients were divided into two groups: indeterminate (*n* = 29, Fig. 1) and positive (*n* = 43, Fig. 2).

Among the 181 patients with negative results, 17 patients relapsed, and 19 died of progressive disease (*n* = 12), infection (*n* = 3), heart failure (*n* = 1), lung cancer (*n* = 1), or unknown causes (*n* = 2) by the end of follow-up.

Among the 29 indeterminate patients, 6 underwent biopsy following F-PET/CT. Of the 6 patients, none were positive, and 4 remained disease-free by the end of follow-up. Among the 29 patients, 9 relapsed, but only 3 died of progressive disease by the end of follow-up.

Among the 43 patients with positive results according to the QVTA criteria, 33 (76.7 %) died by the end of follow-up. The median survival was 23 months.

### Prognosis predictive value of F-PET/CT according to the Deauville criteria

**Fig. 3:**
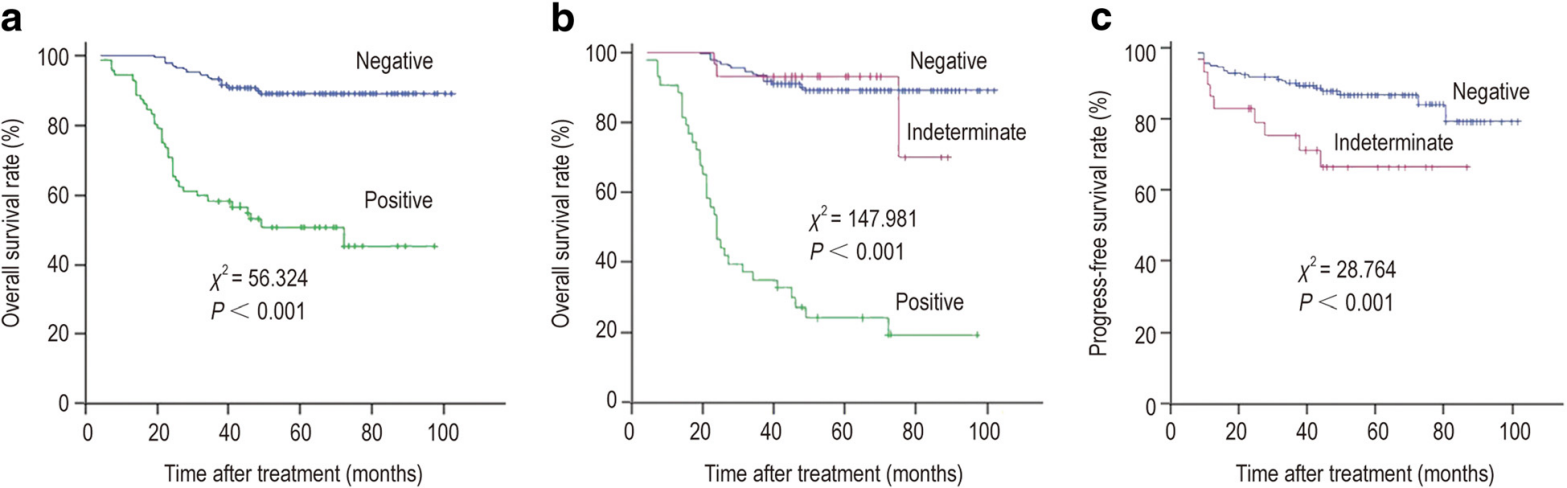
Kaplan-Meier survival curves based on PET/CT findings in 253 patients with DLBCL. (**a**), overall survival (OS) curves of patients with positive (n = 72) and negative (n = 181) F-PET/CT scans according to the Deauville criteria. (**b**), OS curves of patients with positive (n = 43), indeterminate (n = 29), and negative (n = 181) F-PET/CT scans according to the QVTA criteria. (**c**), progression-free survival (PFS) curves of patients with negative (n = 181) and indeterminate (n = 29) F-PET/CT scans according to the QVTA criteria

The 3 year OS rate was 91.6 % (95 % confidence interval [CI], 87.5 %-95.7 %) for patients with negative F-PET/CT scan results and 57.5 % (95 % CI, 46.5 %-68.5 %) for patients with positive results. The 3 year OS rate was significantly higher in patients with negative results than in patients with positive results (chi-square = 56.32, *P* < 0.001) (Fig. 3a).

### Prognosis predictive value of F-PET/CT according to the QVTA criteria

The 3 year OS rate was significantly higher in patients with indeterminate results than in patients with positive results (91.2 % [95 % CI, 87.1 %-95.3 %] vs. 33.5 % [95 % CI, 19.5 %-47.5 %], chi-square = 147.98, *P* < 0.001), but there was no significant difference between patients with negative and indeterminate results (91.6 % vs. 91.2 %, chi-square = 0.01, *P* = 0.921) (Fig. 3b).

The 3 year PFS rate was significantly higher in patients with negative results than in patients with indeterminate results (89.3 % [95 % CI, 84.8 %-93.9 %] vs. 73.1 % [95 % CI, 56.7 %-89.7 %], chi-square = 28.76, *P* < 0.001) (Fig. 3c).

## Discussion

In this study, we assessed the value of F-PET/CT in predicting the prognosis of DLBCL and compared the Deauville criteria and QVTA criteria for interpreting the F-PET/CT scans in a histologically homogeneous series of patients with DLBCL. The present study demonstrated that F-PET/CT can predict long-term prognosis. As previously reported, patients with negative F-PET/CT scans live longer than those with positive F-PET/CT scans [12-14]. In our study, the 3 year OS rate was significantly higher in patients with negative F-PET/CT scan results than in patients with positive results, as interpreted according to the Deauville criteria (91.6 % vs. 57.5 %, *P* < 0.001).

When the QVTA criteria were used, 29 patients with F-PET/CT scans interpreted as positive according to the Deauville criteria were classified as indeterminate. Among them, 9 relapsed, but only 3 died of progressive disease by the end of follow-up. Twenty patients achieved complete remission. Six of the 29 patients underwent biopsy following F-PET/CT, and none were positive. Based on these findings, the F-PET/CT scan results for most of the 29 patients, as interpreted according to the Deauville criteria, were evidently false positive. The 3 year OS rate for the patients with indeterminate results was 91.2 %; their outcomes were more similar to those of the patients with negative results compared with patients with positive results. Although the 3 year PFS rate was lower in patients with indeterminate results than in patients with negative results (73.1 % vs. 89.3 %), there was no significant difference in the 3 year OS rate between patients with negative and indeterminate results (91.6 % vs. 91.2 %).

Patients with positive F-PET/CT scans should undergo more aggressive treatment, including a complete course of chemotherapy with a high-dose radiotherapy or a high-dose therapy such as autologous stem cell rescue. Most of the 29 patients with positive F-PET/CT scans, as interpreted by the Deauville criteria, had false positive results. This means that the aggressive (i.e., toxic) treatment they received was unnecessary. Whether withholding aggressive treatment will affect the long-term survival of such patients remain uncertain; thus, further prospective studies are warranted.

It is possible that the false positive scans were due to ^18^ F-FDG being a marker that lacks high specificity and that is taken up in infectious and inflammatory processes [15, 16]. It is also possible that the use of immunotherapy increased lesion inflammation. Antibody-mediated cellular cytotoxicity and complement activation are important mechanisms of action of rituximab [10, 17]; both processes attract mediators of inflammation to the tumor site. The various uses of rituximab in previous studies in a minority of patients [13, 18] compared with its use in all patients in our study may explain the high rate of ^18^ F-FDG positivity unrelated to tumor activity. In this study, using the QVTA criteria allowed us to greatly reduce the number of false positive interpretations.

DLBCLs are relatively heterogeneous and, in a few cases, can switch their genetic or immunohistochemical phenotype and transform into other lymphomas or carry more than one malignant clone [19]. Therefore, the acquisition of drug resistance in lymphoma is driven in part by the inherent genetic heterogeneity of DLBCL and the instability of the tumor cells. The emergence of acquired chemoresistance prevents the successful treatment of DLBCL [20]. It is expected that most chemosensitive tumor cells are killed, but chemoresistant tumor cells continue to proliferate to form clumps. It is expected that the clumps formed by these chemoresistant tumor cells have the same metabolic activity and show clearly uniform high focal ^18^ F-FDG uptake on PET/CT scans. Based on this assumption, this study considered only centralized and uniform residual mass ^18^ F-FDG distribution as positive.

The value of PET/CT in prognostic prediction for aggressive lymphomas is distinguishing. Although patients with positive F-PET/CT scans, as interpreted by the Deauville criteria, had poorer OS compared with the patients with negative F-PET/CT scans, the 3 year OS rate remained 57.5 %. When the QVTA criteria were applied, only 43 patients were classified as positive. Of these patients, 33 (76.7 %) died by the end of follow-up. The median survival was 23 months. The 3 year OS rate of the patients with positive results decreased to 33.5 %.

Given the retrospective data collection and relative disparity in treatment types, we acknowledge that this study had limitations and potential biases.

Nevertheless, this retrospective study emphasizes that F-PET/CT interpreted using the QVTA criteria is feasible for therapeutic response evaluation and can be used to guide risk-adapted therapy. Compared with the Deauville criteria, the QVTA criteria can distinguish high-risk patients with poor outcomes more accurately. This information may be useful for objectively tailoring risk-adapted consolidation strategies.
